# Anxiety and Depression Comorbidities in Moroccan Patients With Breast Cancer

**DOI:** 10.3389/fpsyt.2020.584907

**Published:** 2021-01-12

**Authors:** Ouassil El kherchi, Amina Aquil, Noureddine El khoudri, Mustapha Mouallif, Mohamed Daghi, Maroua Guerroumi, El madani Saad, Abdellatif Benider, Arumugam R. Jayakumar, Abdeljalil Elgot

**Affiliations:** ^1^Epidemiology and Biomedical Unit, Laboratory of Sciences and Health Technologies, Higher Institute of Health Sciences, Hassan First University of Settat, Settat, Morocco; ^2^Mohammed VI Center for the Treatment of Cancers, Ibn Rochd University Hospital Center Casablanca, Casablanca, Morocco; ^3^Neuropathology Research Unit, Miami VA Medical Center and Department of Obstetrics, Gynecology and Reproductive Sciences University of Miami Miller School of Medicine, Miami, FL, United States

**Keywords:** breast cancer, anxiety, depression, sleep quality, body image, Morocco, survey

## Abstract

**Background:** Breast cancer is the leading cause of cancer death in women worldwide with more than 1. 7 million new cases annually. Major advances have been made in the therapeutic management of this condition in many countries. However, neuropsychiatric disorders in patients with breast cancer constitute a significant concern due to their negative impact on patient's life and on the success of therapy itself.

**Methodology:** In this study we aimed to evaluate psychological disorders in a population of 212 Moroccan women treated for breast cancer within the Mohammed VI Center for the Treatment of Cancers of Casablanca. A questionnaire was designed to this end on the basis of different validated scales, including the Hospital Anxiety Depression Scale (HADS), the Pittsburgh Sleep Quality Index (PSQI), and the Body Image Scale (BIS).

**Results:** Data analysis has shown that 69.3% of participants had significant sleep disorders; 87% suffer from an anxiety-depressive syndrome (ADS), and 83.0% had significant body image dissatisfaction. A positive correlation was shown between ADS and both patients' national health insurance coverage and patients or husbands' education. Analysis further revealed that anxiety and depression were negatively correlated with different types of treatment. Similarly, both BIS and PSQI scores were positively associated with anxiety and depression disorders.

**Conclusion:** The present investigation highlights the need to generalize and strengthen the psychological approach of patients treated for breast cancer in Morocco. We anticipate that such a strategy will alleviate suffering and promote therapy success in these patients and will diminish or prevent conjugal and familial impacts of the illness.

## Introduction

Breast cancer is considered to be one of the most common public health issues in the world. It's the leading cause of mortality due to cancer in women in the world, with more than 1.7 million new cases and ~521,900 deaths each year (1). Various investigations have shown a negative impact of this pathology on the quality of life of patients, due to its chronicity and malignancy (2). In addition, although the various healing forms—chemotherapy, radiotherapy, and surgery—allowed better management of patients suffering from this pathology, they affect the mental health of patients and cause significant mood disorders (3, 4). According to a recent epidemiological study published by a German research team, 32% of people with cancer in general and 42% of those with breast cancer in particular, have a high level of mental disorders and emotional distress (5). These disorders are the main criteria for the Diagnostic and Statistical Manual of mental disorders (DSM-IV), which include, among others, anxiety, and depression. Indeed, new studies suggest that depression is a common comorbidity in cancer patients, who exhibit poor quality of life, more functional disorders, higher levels of suicidal ideation, and significant death rates (6).

In this setting, depression disorders consist of a combination of major depressive disorders, persistent depressive disorders—also known as dysthymic disorders—and a mixed form of both depression and anxiety disorders (7). In addition, women with breast cancer show anxiety, including post-traumatic stress syndrome and generalized anxiety disorders characterized by persistent and excessive worries about numerous events (8). In Morocco, breast cancer is the leading cancer in terms of incidence and mortality (9). The quality of life and psychiatric health care of people with this pathology is gaining more and more interest but has still not reached desirable levels. Hence, in Morocco, very few data are available on this subject. The few published data on Moroccan patients claim that radical mastectomy should be avoided “as far as possible” in young women suffering from breast cancer, as it engenders in them more anxiety and more depression disorders, whereas conservative surgery should be practiced whenever possible for young patients to cope with psychological distress related to mastectomy (10).

The same Moroccan team working at the Moroccan National Institute of Oncology at Rabat, the capital, suggested many interventions that should be done in order to protect patients living with breast cancer against psychological distress, among them, social family support and both emotional and financial facilities (11).

However, a study published by Haddou Rahou and his collaborators highlighted a lack of studies on the quality of life of women with breast cancer in different Muslim and Arab countries, including Morocco. Hence the need to conduct a comprehensive investigation (12).

The aim of this study was to evaluate both anxiety and depression disorders in a population of Moroccan breast cancer patients within the region of Casablanca, the largest Moroccan city, and to identify the anxio-depressive impacts of some socio-demographic parameters and some clinical variables including types of treatment, sleep disturbance, and body image dissatisfaction.

## Methods and Materials

### Study Design and Sample

The present descriptive and cross-sectional analysis was carried out between February 2018 and May 2019 in the Mohammed VI Center for the Treatment of Cancers, Ibn Rochd University Hospital of Casablanca, Morocco. Casablanca is the largest city in Morocco and the economic capital of the country. During this study, 212 breast cancer patients were randomly chosen among patients coming for follow-up at the Oncology Department. The inclusion criteria were the following: women with confirmed diagnosis of breast cancer, those agreeing to participate in this study with written consent (for unschooled patients, verbal consent followed by a fingerprint was used as proof of consent), patients with no previous follow-up of any psychiatric and mental disorders, and the ability to understand and speak the Moroccan dialect. The latter was to ensure efficient and easy communication during the completion of the questionnaire.

### Questionnaire Validation

Before proceeding with data collection using the Hospital Anxiety and Depression Scale (HADS), the Pittsburgh Sleep Quality Index (PSQI), and the Body Image Scale (BIS), two translators translated the questionnaire from English into dialectal Arabic, and then another team made up of two other translators performed a back translation of the questionnaire. The two versions of the questionnaire were studied by a team of experts (translators, a nurse, a psychiatrist, an epidemiologist, and an oncologist) to produce a pre-final version. Finally, a pilot study was carried out on 30 cancer patients to test comprehension of the items. The reliability test calculation (Cronbach α) indicates good validity for the three scales (HADS, BIS, and PSQI).

### Assessment of Anxiety and Depression Disorders

In this study we used a questionnaire that provided participants' socio-demographic data and the Hospital Anxiety and Depression Scale (HADS). The HADS, developed by Zidmond and Snaith (13), assesses the dimensions of anxiety and depression in non-psychiatric populations and largely used in psycho-oncology research. In addition, it offers the advantage of eliminating, at the same time, the physical symptoms that can be bias factors. The HADS consists of two subscales of seven items each, which give two scores, the first measuring anxiety (HAD-A) and the second, depression (HAD-D). The anxiety and depression subscales have three levels: 0–7, absence of anxiety or depressive disorders; from 8 to 10, suspected anxiety or depressive disorders; and from 11 to 21, proven anxiety or depressive disorders. The total HADS-T scale ranges from 0 to 14 (absence of anxio-depressive disorders) and from 15 to 42 (existence of anxio-depressive disorders). The Cronbach α for the HADS scale used in this study is 0.76, showing good validity.

### Assessment of Sleep Quality Disorders

The Pittsburgh Sleep Quality Index (PSQI) used in the questionnaire has 19 items grouped into seven components, including: (i) subjective sleep quality, (ii) sleep latency, (iii) duration of sleep, (iv) habitual sleep efficiency, (v) sleep disturbances, (vii) sleep medication use, and (vii) daytime dysfunction due to lack of sleep. Each component score ranges from 0 to 3, where 3 refers to a severe sleep disturbance. The summation of the PSQI scores yields a total score ranging from 0 to 21. A total score >8 indicates a patient with a significant sleep disorder and poor sleep quality. The Cronbach α for the PSQI in this study is 0.67; this value is strong and approaches one of the original validations (14).

### Assessment of Body Image Dissatisfaction

In the current study, body image dissatisfaction regarding breast cancer patients was assessed using the body image scale (BIS) (15). This scale includes 10 items developed to evaluate briefly and consistently the affective, behavioral, and cognitive aspects of the body image in cancer patients who are undergoing appearance changes. It also reflects the impact of cancer treatment, including surgery, on the body image of breast cancer patients. The BIS components score ranges from 0 (not at all) to 3 (very much), and the BIS final score (the sum of the 10 items subscales) ranges from 0 (body image satisfaction) to 30 (strong body image dissatisfaction). The Cronbach α for this study is 0.85 which demonstrated a very a good reliability of the used questionnaire.

### Statistical Analysis

The descriptive analysis consisted of calculating absolute and relative frequencies for qualitative variables, in addition to positioning and dispersion parameters for quantitative variables (average, standard deviation).

The normal distribution of the variables was examined with the Kolmogorov-Smirnov test. In bivariate analysis, the continuous variable comparison involved the Student *t*-test, the Mann-Whitney test, ANOVA, and the Friedman test.

The significance level was set at *p* < 0.05. The statistical analysis was carried out using SPSS software version 19.0.

## Results

### Socio-Demographic Characteristics

The present study was based on data from a random sample of Moroccan woman with breast cancer. The age range was from 22 to 82 years (*n* = 212), with a mean age of 49.66 (SD = 12.50). About half of the participants (49.53%) and their husbands (56.60%) were unschooled and exhibited a low educational level, and about 52% were of low socioeconomic status. As for insurance coverage, the vast majority of our study population (93.4%) had RAMED, which is a national health insurance program intended to allow low-income Moroccan citizens access to health facilities (Table 1).

**Table 1:** Demographic and clinicopathological characteristics of the breast cancer sample.

**Characteristics**		**Study population (*****n*** **=** **212)**	
		***n***	**%**
Women's age (Years)	<35	21	9.91
	35–49	103	48.58
	>50	88	41.51
Education's level	Unschooled	105	49.53
	Elementary	44	20.75
	High school and university	63	29.72
Socioeconomic status	Low	109	51.42
	Average and high	103	48.58
Health insurance coverage	CNPS CNSS	14	6.60
	RAMED	198	93.40
Residential typologies	Rural	54	25.47
	Urban	158	74.53
Husduand's education	Unschooled	120	56.60
	Elementary	44	20.75
	High school and university	48	22.64
Types of treatments	**(Two)** surgery, chemotherapy	69	32.55
	**(Three)** surgery, chemotherapy, hormone therapy	131	61.79
	**(Four)** surgery, radiotherapy, chemotherapy, and hormone therapy or immunotherapy	12	5.66
BODY Image Scale (BIS)- total score	BIS-S	36	16.98
	BIS-D	176	83.02
Pittsburgh Sleep Quality Index (PSQI-T)	PSQI-T <8	65	30.66
	PSQI-T >8	147	69.34

### Clinical and Neuropsychiatric Characteristics

After analyses of patients' medical history, several treatments were administered to our breast cancer patients. Thus, almost two thirds of our sample (61.79%) received surgery and/or chemotherapy or hormone therapy. In all, 32.55% of patients received only surgery and chemotherapy. A minority of our participants (5.66%) had surgery, radiotherapy, chemotherapy, and hormone therapy together (Table 1).

As for the assessment of sleep quality, the total score of the Pittsburgh Sleep Quality Index (PSQI-T) revealed that more than two thirds of our breast cancer patients (69.34%) had significant sleep disorders and exhibited poor sleep quality (Table 1).

The monitoring of neuropsychiatric parameters revealed that our population sample exhibited severe psychiatric comorbidities. In fact, the HADS and the BIS data analysis showed that 86.67% of our breast cancer patients suffered from an anxiety-depressive syndrome (ADS). Results further showed that almost 83% our population suffered from not being attractive and then expressed significant body image dissatisfaction (BIS-D) (Table 1).

### Correlation Between the HADS Components and Both Socio-Demographic and Clinical Characteristics

In this study, three components of the HADS scale were used: HADS-A, for anxiety; HADS-D, for depression; and HADS-T, for anxio-depressive syndrome. The three components allowed the assessment of the presence or absence of these neuropsychiatric disorders, which usually occur in women with breast cancer (Table 2).

**Table 2:** Comparison between the Hospital Anxiety and Depression scale components (HADS-A, HADS-D, HADS-T) and both demographic and clinicopathological characteristics.

**Characteristics**		**HADS-A**		**HADS-D**		**HADS-T**	
		**Mean ± SD**	***P*-value**	**Mean ± SD**	***P*-value**	**Mean ± SD**	***P*-value**
Women's age (Years)	<35	12.67 ± 4.041	0.29	10.38 ± 4.353	0.35	23.05 ± 7.194	0.31
	35–49	13.96 ± 3.559		10.99 ± 3.974		25.05 ± 6.82	
	>50	13.88 ± 3.293		11.56 ± 3.328		25.43 ± 5.669	
Educational levels	Unschooled	14.54 ± 3.337	0.0001^***^	11.95 ± 3.341	0.0001^***^	26.5 ± 5.696	0.0001^***^
	Elementary	14.18 ± 2.326		11.7 ±0.764		25.89 ± 5.226	
	High school and university	12.29 ± 4.002		9.48 ± 3.926		21.92 ± 7.247	
Socioeconomic status	Low	14.08 ± 3.588	0.22	11.72 ± 4.028	0.028^*^	25.8 ± 6.8	0.065
	Average and high	13.05 ± 3.407		10.58 ± 3.377		24.17 ± 5.89	
Health insurance coverage	CNPS CNSS	15.71 ± 2.4	0.034^*^	11.36 ± 2.341	0.84	27.07 ± 2.674	0.014^*^
	RAMED	13.66 ± 3.535		11.15 ± 3.844		24.86 ± 6.575	
Residential typologies	Rural	14.28 ± 3.153	0.24	11.91 ± 3.338	0.093	26.19 ± 5.624	0.12
	Urban	13.63 ± 3.612		10.91 ± 3.871		24.61 ± 6.627	
Husband's education	Unschooled	14.61 ± 2.979	0.097	11.89 ± 3.198	0.05^*^	26.5 ± 5.168	0.05^*^
	Elementary	13.36 ± 3.807		10.4 ± 5.058		23.76 ± 8.243	
	High school and university	13.3 ± 3.941		10.2 ± 3.624		23.83 ± 6.953	
Types of treatments	Two	14.29 ± 3.01	0.008^**^	11.42 ± 3.371	0.005^**^	25.71 ± 5.196	0.002^**^
	Three	13.8 ± 3.535		11.34 ± 3.843		25.22 ± 6.582	
	Four	10.92 ± 4.621		7.75 ± 3.596		18.67 ± 7.866	
Body Image Scale (BIS)- total score	BIS-S	12.08 ± 3.667	0.001^**^	9.19 ± 4.341	0.004^**^	21.28 ± 6.606	0.0001^***^
	BIS-D	14.15 ± 3.376		11.57 ± 3.905		25.77 ± 6.114	
Pittsburgh Sleep Quality IndeX (PSQI-T)	PSQI-T <8	12.82 ± 3.863	0.007^**^	10.54 ± 3.657	0.64	23.28 ± 6.677	0.009^**^
	PSQI-T>8	14.22 ± 3.258		11.48 ± 3.773		25.78 ± 6.159	

* *p < 0.05*,

** *p < 0.001*,

*** *p < 0.0001*.

Throughout the present study, we noticed that our general population showed suspected or well-proven anxiety and/or depression. The suspected anxiety or depression is assessed from a HADS-A or HADS-D score from 8 to 10. Anxiety or depressive disorders are proven by HADS-A or HADS-D scores ranging from 11 to 21. Data also indicate the presence of anxio-depressive syndrome (ADS) for the whole of our breast cancer population (total score, HADS-T >15) (Table 2). According to our results, anxiety and depressive disorders could affect any age range without any distinction (*p* > 0.05). However, a strong correlation was found between educational levels and the onset of both anxiety and depression in our sample (*p* = 0.0001). This correlation was also seen between the husband's education levels and both depression and ADS (*p* = 0.05).

Furthermore, data indicate no association between the aforementioned neuropsychiatric and socioeconomic status (*p* > 0.05), whereas our results showed a noticeable association between national health insurance coverage (RAMED) and both anxiety disorder (*p* = 0.034) and ADS (*p* = 0.014) (Table 2).

As for the effect of other health factors on the mental health of our breast cancer population study, results showed a strong association between the number of treatments, sleep quality (PSQI-T score), and body image dissatisfaction (BIS-D score). In light of this, different types of treatments received by our patients, including surgery, chemotherapy, radiotherapy, hormone therapy, and immunotherapy, had a negative effect on the mental health of our study population, which exhibited a strong association with anxiety disease (*p* = 0.0008), depression (*p* = 0.0005) and ADS (*p* = 0.0002) (Table 2).

In terms of the impact of sleep quality on the mental health of patients with breast cancer, our data indicate a strong correlation between the Pittsburgh index (PSQI-T ≥ 8) and both anxiety disease (*p* = 0.0007) and ADS (*p* = 0.0009) (Table 2).

Our results show also that the psychological disturbances revealed in this study are strongly related to the side effects of the surgery seen on patients' bodies. Thus, the assessment of body image dissatisfaction (BIS-D) exhibits solid association with anxiety (*p* = 0.001), depression (*p* = 0.004), and ADS (*p* = 0.0001).

## Discussion

Anxiety and depression are the main mental health disorders directly or indirectly induced either by breast cancer, its treatment, or only its disclosure during the first diagnosis. The psychological distress is the term generally adopted in oncology and cancer care to refer to these two mental disorders, which affect more than one third of breast cancer patients (16). In Morocco as well as in other developing countries, few studies have focused on the evaluation of the psychological component of patients with breast cancer. From this point of view, efforts should be made to improve adapted health care against anxiety and depression for breast cancer. To the best of our knowledge, this study is the first to examine the possible link between the anxio-depressive syndrome, sleep disturbance, and body image dissatisfaction in a population of Moroccan patients with breast cancer.

In this study, all breast cancer patients received several treatments, but chemotherapy and surgery were the first to be practiced. According to our results, these medical interventions induced anxiety, depression, and ADS. Our findings are in agreement with data from the literature showing that cancer treatments worsen the quality life of breast cancer patients and lead to the deterioration of their mental health, mainly manifesting itself as anxiety and symptoms of depression (3, 4, 17). Moreover, another study suggested that anxiety remains persistent during treatment, which could likely be related to misunderstanding the efficiency of therapeutic approaches used in breast cancer treatment (18). Furthermore, it has been shown that depression decreases the incentive for chemotherapy and lowers the compliance with both chemotherapy and radiotherapy, leading to cancer progression and reducing patients' lifespan (19, 20).

In our breast cancer sample, it seems that anxiety and depression comorbidities are strongly correlated to patients' body image dissatisfaction. The latter comes from visible body modification, such as breast and hair losses, and stems also from perceptible manifestations such as hot flashes. Our finding is in agreement with recent studies showing that persistent body image distress is closely linked to psychological distress, such as anxio-depressive syndrome (21, 22). Other studies confirm that 33% of breast cancer survivors experiencing body image dissatisfaction still have this feeling for up to 9 years post-surgery (23, 24). From this point of view, it is well-known that depression is a serious mental disease that can cause severe feelings of sadness and hopelessness that could lead breast cancer patients to criticize themselves after breast loss, which leads to high levels of body image dissatisfaction. To deal with this, self-compassion and hope-focused therapy may be useful to relieve body image dissatisfaction of patients with breast cancer (25). Further, many others suggestions could be brilliants alternatives. Thus, the focus on couples or groups within clinic and psychological settings seems to be promising (23), even though such interventions could have limited effectiveness for younger breast cancer patients (22) and require more health professionals and financially expensive treatment (26). In our context, such an approach needs incentives and subsidies granted by the Ministry of Health, since the majority of breast cancer patients attending the public hospitals are from low-income backgrounds. In our population study, more than half of the patients had a low socioeconomic status and did not have the ability to enter private health care. Moreover, the majority of our sample had RAMED, which is a national health insurance. To cope with this socioeconomic issue and in order to correct the body image dissatisfaction of breast cancer patients experiencing psychological distress, new strategies are being used in oncology (27). One of them consists of unstructured writing activities (23), which aim to enhance the confidence and self-appreciation for mastectomy patients. Along these lines, psychologists from Macquarie University of Sydney have developed a program called “My Changed Body,” which is a web platform containing writing exercises intended to encourage self-compassion and to lower anxiety and depression related to the body image dissatisfaction (28).

The extrapolation of this strategy to our sample appears infeasible because of the high level of illiteracy. In another context, our results displayed a significant impact of sleep quality disturbance on the mental health of our population study. Thus, a strong correlation between the Pittsburgh index and both anxiety disorder and ADS was found. We can confirm that our results are in agreement with those published by other researchers (29). Moreover, it has been shown not only in breast cancer but in other types of cancer that sleep disturbance is accompanied usually by noticeable fatigue, depressive symptoms, and anxiety disorders (30–32). Those previous findings show a strong association between sleep disturbance and both fatigue and depression (33, 34). In light of this, Fiorentino and Ancoli-Israel claim that breast cancer patients usually complain of sleep disorders with a percentage ranging between 20 and 70% (17). Moreover, sleep disturbance rates are found to be higher in women with breast cancer than in people with other types of cancer as well as in people with non-cancerous conditions (35). From the analysis of our results and comparisons of them with others, it appears that sleep disturbance in breast cancer patients is highly correlated with most types of treatments especially surgery, chemotherapy, and radiotherapy (32). The pathophysiological mechanism by which chemotherapy induces sleep disturbance remains unclear, but some researchers have suggested that proinflammatory cytokines and circadian rhythm perturbation could be the basis for the sleeplessness observed in breast cancer patients undergoing chemotherapy (32). Additionally, breast cancer patients who have completed chemotherapy treatment exhibit less sleep depth, less Rapid Eye Movement sleep (REM), and less sleep effectiveness compared with people without cancer (36).

Concerning the most useful interventions for the relief of sleep disturbances and fatigue in patients with breast cancer undergoing radiotherapy treatment, Mock and collaborators assessed the impact of home-based walking exercise (4–5 days/week, 20–30 min/day over 6 weeks). The results show a significant improvement of sleep quality as well as in relief of anxiety and fatigue (37).

## Conclusion

Overall, breast cancer is known to be an alarming disease that causes women both physical and psychological suffering. In this study we have demonstrated that our population exhibited severe psychiatric comorbidities, especially anxiety and depression. Besides low socioeconomic status and low educational level, we found that most types of treatments, sleep disorders, and body image dissatisfaction encountered by women with breast cancer exacerbate both anxiety and depression levels inducing in patients a worse psychological health status.

From this study, it seems that the pathological triad of anxio-depressive syndrome, sleep disorders, and body image dissatisfaction forms a kind of vicious circle and that each of them potentiates the effects of the others. When these three comorbidities are associated in the same breast cancer patients, the patients' survival rates decrease.

Given the results of this study, we recommend that health care providers and decision-makers in the field of health strengthen psychological care teams for breast cancer patients and consider establishing mobile psychological health care units in order to visit patients in their homes, especially those living in isolated areas or with low socioeconomic status.

This study has some limitations. On the one hand, our sample may not be representative of the general population of Moroccan women with breast cancer, due to the recruitment of a limited sample in a single counter hospital. On the other hand, our work is conducted only at the university hospital center, which welcomes patients coming from both urban and rural areas of the Casablanca-Settat region, the country's economic capital. For future studies, we plan to initiate a multicentric study which would include other hospital universities and also the private sector.

## Data Availability Statement

The original contributions presented in the study are included in the article/supplementary materials, further inquiries can be directed to the corresponding author/s.

## Ethics Statement

The studies involving human participants were reviewed and approved by Mohammed VI Center for the Treatment of Cancers, Ibn Rochd University Hospital Center Casablanca, Morocco approved the study presented here. The patients/participants provided their written informed consent to participate in this study.

## Author Contributions

OE, AA, and AE: conception and design of the study. MD, AA, and MG: data collection. NE, AA, OE, and ES: acquisition and data analysis. AB: recruitment of patients. OE, MM, AJ, and AE: interpretation of data. OE and AA drafting of the work. MM, AB, AJ, and AE: revising the manuscript critically and final approval of the manuscript. All authors: approved the final version of the manuscript to be submitted.

## Conflict of Interest

The authors declare that the research was conducted in the absence of any commercial or financial relationships that could be construed as a potential conflict of interest.
